# Effects of branched-chain amino acid granules on serum albumin level and prognosis are dependent on treatment adherence in patients with liver cirrhosis

**DOI:** 10.1111/j.1872-034X.2012.01097.x

**Published:** 2012-10-10

**Authors:** Koichi Takaguchi, Hisataka Moriwaki, Hisashi Doyama, Masayuki Iida, Michiyasu Yagura, Noritomo Shimada, Masahiro Kang, Haruki Yamada, Hiromitsu Kumada

**Affiliations:** 1 Department of Hepatology, Kagawa Prefectural Central HospitalKagawa; 2 Department of Medicine, Gifu University Graduate School of MedicineGifu; 3 Department of Gastroenterology, Ishikawa Prefectural Central HospitalKanazawa; 4 Department of Internal Medicine, Nagoya Midori Municipal HospitalNagoya; 5 Department of Gastroenterology, National Hospital Organization Tokyo National HospitalTokyo; 6 Department of Internal Medicine, Social Insurance Chuo General HospitalTokyo; 7 Department of Hepatology, Toranomon HospitalTokyo; 8 Department of Internal Medicine, Medical Plaza Heiwadai HospitalChiba; 9 Department of Internal Medicine, Sato Daiichi HospitalOita, Japan

**Keywords:** branched-chain amino acids, hepatic failure, liver cirrhosis, prognosis, serum albumin, treatment adherence

## Abstract

**Aim:**

To test if the treatment adherence to branched-chain amino acid (BCAA) granules influences the serum albumin level and prognosis in prospective 2984 patients with decompensated liver cirrhosis who were prescribed BCAA granules containing 952 mg of L-isoleucine, 1904 mg of L-leucine and 1144 mg of L-valine at 4.15 g/sachet three times a day after meals.

**Methods:**

The primary end-point was the time to the event defined as “hospital admission due to progression of hepatic failure”, and factors affecting this outcome were explored. Changes in serum albumin level were evaluated as the secondary end-point.

**Results:**

Patients were divided into the good adherence group (those who reported to have taken “nearly all” prescribed doses) and the poor adherence group (those who reported to have taken “approximately half” or “less” doses), because such stratification was validated by treatment responses in plasma BCAA/tyrosine ratio. Factors related to the primary end-point were age, drug adherence during 6 months of study treatment, previous hepatic cancer, current clinical manifestations, previous clinical manifestations, baseline serum albumin level, platelet count and total bilirubin level. The cumulative event-free survival was significantly higher in the good adherence group. Increase in the serum albumin level was also greater in the good adherence group.

**Conclusion:**

Higher BCAA treatment adherence better raised the serum albumin level, leading to improvement of event-free survival. These results indicate the importance of patient instruction for the adequate use of BCAA granules.

## Introduction

ALTHOUGH LIVER CIRRHOSIS is caused by any of a wide variety of etiologies,^1^ clinical features of the disease share complications such as ascites, edema, hepatic encephalopathy and esophageal varices.^2^ Some of these complications are attributable to decreased serum concentrations of albumin and other proteins,^3^–^5^ and oral supplemental branched-chain amino acid (BCAA) therapy with BCAA granules or BCAA-enriched nutrients is recommended, in addition to dietary treatment with adequate protein and energy intake, for the management of these complications.^6^–^8^

Branched-chain amino acid granules are used for the improvement of hypoalbuminemia in patients with decompensated liver cirrhosis,^4^,^9^–^11^ and several studies have demonstrated their efficacy in reducing complications of liver cirrhosis.^4^,^12^–^14^ Furthermore, a reduction in the risk of hepatic cancer is also reported in patients taking BCAA granules.^12^,^15^–^17^

On the other hand, the patients’ treatment adherence was not so favorable owing to the size of individual doses and unpleasant taste, causing interruption of treatment^13^ or reduction of doses.^4^ Although serum albumin level has been shown to improve in a dose-dependent manner based on the prescribed BCAA doses,^10^ no studies have investigated exactly how treatment adherence may influence the serum albumin level and prognosis of patients with liver cirrhosis.

We conducted the present analysis to evaluate how treatment adherence may affect the serum albumin level and prognosis in a prospective cohort of 5042 patients with liver cirrhosis who had started BCAA treatment at a fixed dose of three sachets/day in a preceding study.^18^

## Methods

### Study design and protocol

THIS WAS A multicenter prospective observational study to determine the incidence of adverse events, including hepatocellular carcinoma (HCC) and cirrhosis-related events, under the actual condition of treatment in patients with decompensated liver cirrhosis who were prescribed BCAA granules between June 2003 and December 2006,^18^ and were further followed up thereafter.

A total of 5042 patients with decompensated liver cirrhosis, who presented hypoalbuminemia despite adequate dietary intake, were enrolled in this study at 929 medical institutions in Japan. These patients were p.o. administrated BCAA granules containing 952 mg of L-isoleucine, 1904 mg of L-leucine and 1144 mg of L-valine (Livact Granules, Ajinomoto Pharmaceutical, Tokyo, Japan) at 4.15 g/sachet three times a day after meals.

**Figure 1:**
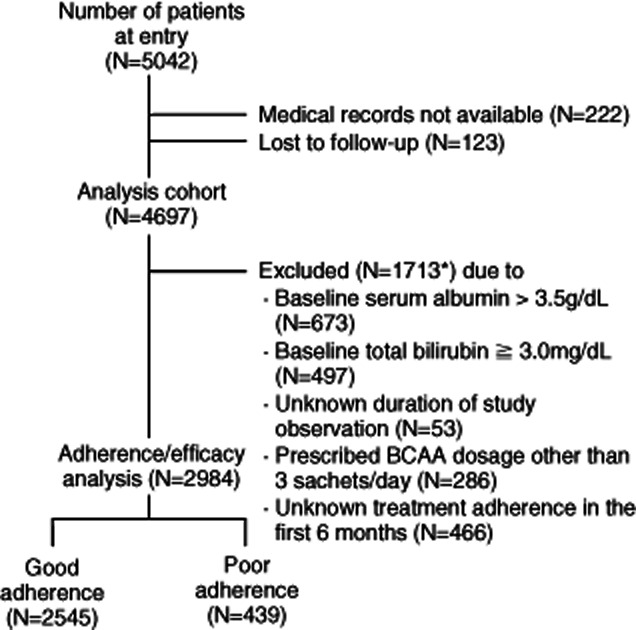
Patient flow. *Among the 1713 patients, 262 were excluded by meeting two or more conditions of the exclusion criteria. BCAA, branched-chain amino acid.

Patient flow is shown in Figure 1. Of the 5042 patients enrolled, the medical records were not available for 222 patients, and 123 patients were lost to follow up after the initial hospital visit. Thus, the remaining 4697 patients constituted the prospective cohort. Patients meeting any of the following criteria were then excluded, and the remaining 2984 patients were subjected to the analysis: (i) a baseline serum albumin level higher than 3.5 g/dL; (ii) a baseline serum total bilirubin level of 3.0 mg/dL or higher; (iii) unknown duration of study observation; (iv) baseline dosage of prescribed BCAA granules other than three sachets/day; or (v) unknown BCAA treatment adherence for 6 months after the start of study observation.

The patients’ treatment adherence was evaluated by a questionnaire analysis at the end of the 6-month surveillance period. The questionnaire provided three answer arms who took “nearly all”, “approximately half” and “less” of the prescribed dose of BCAA granules at three sachets/day. Each patient was instructed to select one of the above three answer arms that best reflected his/her drug adherence status in the preceding study period.

The primary end-point was the time to onset of the event, defined as hospital admission due to progression of hepatic failure, including ascites, edema, jaundice and hepatic encephalopathy. Changes in liver function during the 6 months were evaluated as the secondary end-point.

This study was conducted in accordance with the Japanese Good Post-Marketing Surveillance Practice.

### Statistical analysis

Continuous data were expressed as mean ± standard deviation, and differences in mean values were statistically tested using paired or unpaired Student's *t*-test as appropriate. Categorical variables were compared by Wilcoxon signed rank test, Wilcoxon rank sum test or χ^2^-test as required. The cumulative event-free survival rates were estimated using the Kaplan–Meier method and compared by log–rank test. Any risk factors contributing to the primary end-point were investigated by univariate and multivariate analyses using a Cox proportional hazards model. Data analysis was performed using JMP ver. 9.02 and SAS ver. 9.2 (both SAS Institute, Cary, NC, USA). The level of significance was assessed as two-sided *P* < 0.05.

## Results

### Patients’ characteristics and flow

OF THE PROSPECTIVE cohort consisting of 4697 patients, 1713 were excluded by meeting the exclusion criteria (Fig. 1). Among them, 673 patients had a baseline serum albumin level higher than 3.5 g/dL, 497 patients had a baseline serum total bilirubin level of 3.0 mg/dL or higher, 53 patients had an unknown duration of study observation, 286 patients were prescribed BCAA granules of a dosage other than three sachets/day, and 466 patients reported unknown treatment adherence during the 6 months of study observation. Two hundred and sixty-two patients were excluded by fulfilling two or more conditions of the exclusion criteria. Thus, the remaining 2984 patients were subjected to the adherence/efficacy analysis (Fig. 1). Clinical characteristics of these patients are shown in Table 1. The observation period ranged 6.0–47.9 months, with a median of 21.6 months.

**Table 1:** Clinical characteristics of patients

Characteristics		*n* = 2984
Sex	Male	1584 (53.1%)
	Female	1400 (46.9%)
Age (years)	20–29	1 (0.0%)
	30–39	24 (0.8%)
	40–49	165 (5.5%)
	50–59	530 (17.8%)
	60–69	1038 (34.8%)
	70–79	1024 (34.3%)
	80–89	195 (6.5%)
	>90	7 (0.2%)
	Mean ± SD	66.1 ± 10.1
Cause of liver cirrhosis	HBV	217 (7.3%)
	HCV	1755 (58.8%)
	Alcohol	487 (16.3%)
	PBC	74 (2.5%)
	AIH	63 (2.1%)
	HBV + HCV	16 (0.5%)
	HBV + alcohol	29 (1.0%)
	HCV + alcohol	92 (3.1%)
	HBV + HCV + alcohol	2 (0.1%)
	Other	57 (1.9%)
	Unknown	192 (6.4%)
Treatment adherence (during 6 months)	All	2545 (85.3%)
	Half or less	439 (14.7%)
Previous hepatic cancer	Yes	504 (16.9%)
	No	2454 (82.2%)
	Unknown	26 (0.9%)
Current clinical manifestations	Yes	1568 (52.6%)
	No	1410 (47.3%)
	Unknown	6 (0.2%)
Previous clinical manifestations	Yes	1291 (43.3%)
	No	1670 (56.0%)
	Unknown	23 (0.8%)
Diabetes	Yes	536 (18.0%)
	No	2448 (82.0%)
Serum albumin (g/dL)		3.04 ± 0.36
Platelet (×10 000/μL)		9.73 ± 6.15
AST (IU/L)		67.1 ± 62.8
ALT (IU/L)		47.8 ± 40.9
Serum total bilirubin (mg/dL)		1.30 ± 0.62
BTR		2.95 ± 1.37

For categorical variables, the number of patients and percentage are shown. For continuous variables, the mean ± SD is presented.

AIH, autoimmune hepatitis; ALT, alanine aminotransferase; AST, aspartate aminotransferase; BTR, branched-chain amino acid/tyrosine ratio; HBV, hepatitis B virus; HCV, hepatitis C virus; PBC, primary biliary cirrhosis; SD, standard deviation.

### Risk factors for the primary end-point

For the primary end-point, univariate and multivariate analyses using a Cox proportional hazards model identified the following independent factors to influence the development of the event: age, treatment adherence for the 6 months of study observation, previous hepatic cancer, current clinical manifestations, previous clinical manifestations, baseline serum albumin level, platelet count and serum total bilirubin level (Table 2).

**Table 2:** Risk factors for the event

Explanatory variable		Univariate				Multivariate			
	
		Hazard ratio	*P*-value	95% CI		Hazard ratio	*P*-value	95% CI	
	
				Lower limit	Upper limit			Lower limit	Upper limit
Sex	Male/female	1.18	0.0625	0.99	1.40	1.18	0.0685	0.99	1.42
Age (years)		1.01	0.0190	1.00	1.02	1.02	<0.0001	1.01	1.03
Cause of liver cirrhosis	HBV (yes/no)	1.01	0.9673	0.74	1.34				
	HCV (yes/no)	0.86	0.0928	0.72	1.03				
	Alcohol (yes/no)	1.16	0.1775	0.93	1.42				
Treatment adherence (during 6 months)	Half or less/all	1.74	<0.0001	1.39	2.15	1.94	<0.0001	1.54	2.42
Previous hepatic cancer	Yes/no	1.53	<0.0001	1.25	1.86	1.76	<0.0001	1.42	2.16
Current clinical manifestations	Yes/no	2.21	<0.0001	1.84	2.65	1.66	<0.0001	1.36	2.04
Previous clinical manifestations	Yes/no	1.88	<0.0001	1.59	2.24	1.45	<0.0001	1.20	1.74
Diabetes	Yes/no	1.24	0.0488	1.00	1.52				
Serum albumin (g/dL)	Lower level	2.51	<0.0001	2.02	3.10	2.00	<0.0001	1.57	2.54
Platelet (×10 000/μL)	Lower level	1.04	<0.0001	1.02	1.06	1.03	0.0010	1.01	1.05
AST (IU/L)	Higher level	1.00	0.8840	1.00	1.00				
ALT (IU/L)	Higher level	1.00	0.0156	0.99	1.00				
Serum total bilirubin (mg/dL)	Higher level	1.68	<0.0001	1.47	1.92	1.49	<0.0001	1.29	1.72
BTR	Lower level	1.22	0.0839	0.98	1.59				

Univariate and multivariate analyses were performed using a Cox proportional hazards model, and hazard ratios, *P*-values and 95% CI of the hazard ratios are shown. For the multivariate analysis, variables were selected and determined by backwards selection (*P* = 0.2) using a model incorporating all factors except BTR. BTR was excluded from the multivariate analysis because a considerable proportion of patients lacked BTR data.

ALT, alanine aminotransferase; AST, aspartate aminotransferase; BTR, branched-chain amino acid/tyrosine ratio; CI, confidence interval; HBV, hepatitis B virus; HCV, hepatitis C virus.

### Treatment adherence and plasma BCAA/tyrosine ratio

All these variables except treatment adherence have already been documented as risk factors in patients with liver cirrhosis.^19^,^20^ Taking notice of treatment adherence, therefore, 2545 patients who reported to have taken “nearly all” the prescribed doses during the 6-month period comprised the good adherence group and 439 patients who reported to have taken “approximately half” or “less” of the prescribed doses during that period comprised the poor adherence group for further analysis.

As treatment adherence was judged based on patients’ self-reports, we further attempted to validate the treatment adherence by changes in the BCAA/tyrosine ratio (BTR) as an indicator reflecting true BCAA treatment adherence. Although the number of patients with BTR data was limited (*n* = 185 and 19, respectively), both absolute BTR and relative increase in BTR (increase in BTR/baseline BTR) were higher in the good adherence group (absolute BTR, 4.26 ± 0.65 for the good adherence group and 3.79 ± 0.52 for the poor adherence group; and relative increase in BTR, 0.53 ± 0.8 for the good adherence group and 0.30 ± 0.68 for the poor adherence group; *P* < 0.1 for both) at 6 months of treatment, while there was no significant difference in baseline BTR between the two groups (2.94 ± 0.49 and 2.86 ± 0.46). A comparison between the two groups was thus considered to be feasible.

### Treatment adherence and event-free survival

**Figure 2:**
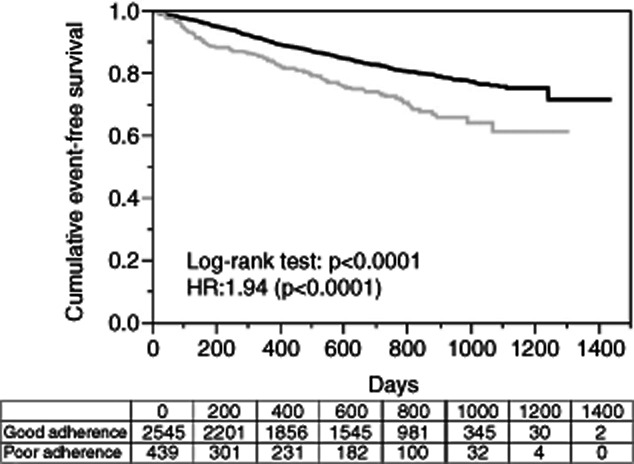
Comparison of cumulative event-free survival rate by treatment adherence status. Cumulative event-free survival rates were estimated for the good adherence and poor adherence groups using the Kaplan–Meier method, and are shown along with the number of patients at risk. Two curves were compared by log–rank test, and hazard ratio (HR) was calculated by Cox proportional hazards model. (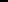) Good adherence; (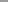) poor adherence.

Regarding the primary end-point, Kaplan–Meier analysis and log–rank test showed a significantly higher cumulative event-free survival rate for the good adherence group as compared with the poor adherence group (Fig. 2).

### Treatment adherence and blood biochemistry

**Figure 3:**
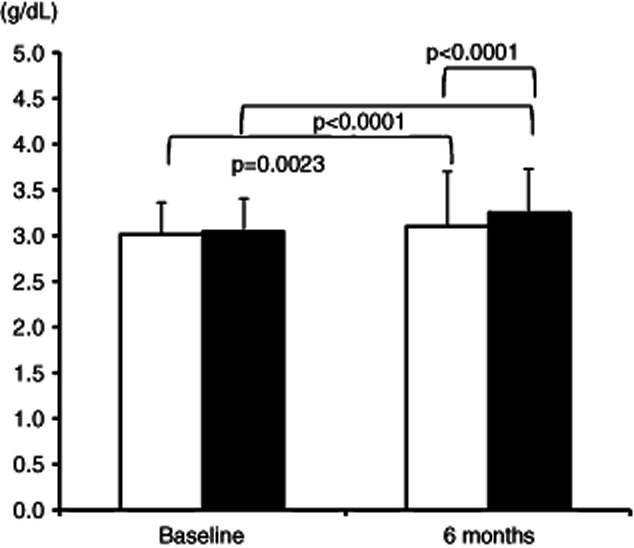
Comparison of serum albumin levels by treatment adherence status. Columns and bars indicate mean and standard deviation of serum albumin levels obtained at baseline and at 6 months of study treatment, respectively. Statistical assessment within each adherence group was carried out by paired Student's *t*-test. For differences between the groups at baseline and at 6 months, Student's *t*-test was conducted. (□) Poor adherence (*n* = 366); (▪) good adherence (*n* = 2378).

Changes in liver function-related parameters during 6 months of the study treatment were examined for each of the good adherence and poor adherence groups. No significant difference was noted in platelet count, aspartate aminotransferase or alanine aminotransferase (ALT) between these groups. At 6 months of study treatment, serum total bilirubin level significantly increased in the poor adherence group but not in the good adherence group. Serum albumin level rose significantly in both of these groups at 6 months of study treatment, and the increase was significantly greater for the good adherence group (Fig. 3).

### Comparison of clinical characteristics between good adherence group and poor adherence group

Baseline clinical characteristics were compared between the good adherence group and poor adherence group as shown in Table 3. Patients of the poor adherence group showed a significantly younger age, lower proportion of hepatitis C virus positivity and higher proportion of alcoholic cirrhosis, lower incidence of previous hepatic cancer, and higher platelet count (Table 3). Also, they tended to be male patients with lower serum ALT activity (Table 3).

**Table 3:** Clinical characteristics of patients by adherence status

Characteristics		Good adherence, *n* = 2545	Poor adherence, *n* = 439	*P*-value
Sex	Male	1334 (52.4%)	250 (56.9%)	*P* = 0.0789
	Female	1211 (47.6%)	189 (43.1%)	
Age (years)	20–29	1 (0.0%)	0 (0.0%)	
	30–39	16 (0.6%)	8 (1.8%)	
	40–49	135 (5.3%)	30 (6.8%)	
	50–59	445 (17.5%)	85 (19.4%)	
	60–69	894 (35.1%)	144 (32.8%)	
	70–79	888 (34.9%)	136 (31.0%)	
	80–89	161 (6.3%)	34 (7.7%)	
	>90	5 (0.2%)	2 (0.5%)	
	Mean ± SD	66.3 ± 9.9	65.2 ± 11.1	*P* = 0.0344
Cause of liver cirrhosis	HBV	184 (7.2%)	33 (7.5%)	*P* = 0.0111
	HCV	1539 (60.5%)	216 (49.2%)	
	Alcohol	393 (15.4%)	94 (21.4%)	
	PBC	59 (2.3%)	15 (3.4%)	
	AIH	52 (2.0%)	11 (2.5%)	
	HBV + HCV	13 (0.5%)	3 (0.7%)	
	HBV + alcohol	23 (0.9%)	6 (1.4%)	
	HCV + alcohol	77 (3.0%)	15 (3.4%)	
	HBV + HCV + alcohol	2 (0.1%)	0 (0.0%)	
	Other	46 (1.8%)	11 (2.5%)	
	Unknown	157 (6.2%)	35 (8.0%)	
Previous hepatic cancer	Yes	448 (17.6%)	56 (12.8%)	*P* = 0.0110
	No	2078 (81.7%)	376 (85.6%)	
	Unknown	19 (0.7%)	7 (1.6%)	
Current clinical manifestations	Yes	1321 (51.9%)	247 (56.3%)	*P* = 0.1545
	No	1218 (47.9%)	192 (43.7%)	
	Unknown	6 (0.2%)	0 (0.0%)	
Previous clinical manifestations	Yes	1094 (43.0%)	197 (44.9%)	*P* = 0.6969
	No	1432 (56.3%)	238 (54.2%)	
	Unknown	19 (0.7%)	4 (0.9%)	
Diabetes	Yes	457 (18.0%)	79 (18.0%)	*P* = 0.9844
	No	2088 (82.0%)	360 (82.0%)	
Serum albumin (g/dL)		3.04 ± 0.36	3.01 ± 0.35	*P* = 0.1519
Platelet (×10 000/μL)		9.56 ± 5.85	10.76 ± 7.63	*P* = 0.0002
AST (IU/L)		67.2 ± 66.1	66.2 ± 38.1	*P* = 0.7578
ALT (IU/L)		48.4 ± 42.7	44.2 ± 28.7	*P* = 0.0518
Serum total bilirubin (mg/dL)		1.29 ± 0.61	1.33 ± 0.66	*P* = 0.2430
BTR		2.98 ± 1.42	2.82 ± 1.07	*P* = 0.4400

For categorical variables, the number of patients and percentage are shown. For continuous variables, the mean ± SD is presented. Statistical analysis was conducted by χ-test or by Student's *t*-test.

AIH, autoimmune hepatitis; ALT, alanine aminotransferase; AST, aspartate aminotransferase; BTR, branched-chain amino acid/tyrosine ratio; HBV, hepatitis B virus; HCV, hepatitis C virus; PBC, primary biliary cirrhosis; SD, standard deviation.

## Discussion

THE LOTUS STUDY demonstrated that the outcome of patients with advanced liver cirrhosis was improved by the treatment with BCAA granules at three sachets/day, compared with the dietary treatment.^4^ As utilized in that study, the recommended dosage of BCAA granules is one sachet three times a day p.o. after meals; however, some patients may not take all three sachets in a day due to problems such as treatment adherence. We therefore conducted the present prospective cohort study to examine how differences in the actual intake of BCAA granules may influence the prognosis of patients with liver cirrhosis.

Assessment of clinical characteristics of the patients included in the present study indicated that these patients shared average clinical features of liver cirrhosis in Japanese patients such as accountable etiologies.^1^ Logistic analysis revealed that none of these causes was an independent risk factor for patients’ outcome. Indeed, the prognosis of patients with liver cirrhosis was determined by eight factors including treatment adherence, regardless of the cause of liver cirrhosis (Table 2).

We focused on the treatment adherence among the eight independent risk factors in the present study, because the clinical significance of the other seven factors has already been described.^19^,^20^ For this concern, patients were divided into the good adherence group (those who reported to have taken “nearly all” prescribed doses) and the poor adherence group (those who reported to have taken “approximately half” or “less” doses), because such stratification was validated by treatment responses in plasma BCAA/tyrosine ratio. Actually, 85.3% of patients reported to have taken “nearly all” three sachets of BCAA granules/day as prescribed. This result was comparable to the 86% adherence in the patients of the LOTUS study.^4^ In the present study, treatment adherence was monitored longer after the first 6 months continuously, and remained similar: 81.1% for 7–12 months, 80.6% for 13–18 months and 79.7% for 19–24 months. These data indicate that treatment adherence observed for the first 6-month period was kept over longer treatment periods and, therefore, suggest that it is reasonable to monitor the treatment adherence of the first 6-month period for the long-term prognosis.

Improvement of hypoalbuminemia was reported to depend on the prescribed daily BCAA doses (8, 12 or 16 g),^10^ but the present study first showed that, at the fixed prescribed dose (three sachets or 12 g/day), serum albumin level rose sufficiently only when the patient had good adherence (Fig. 3). Thus, good treatment adherence resulted in an improved serum albumin level (Fig. 3), and, consequently brought about a higher event-free survival (Fig. 2), as a decreased serum albumin level was also an independent risk factor for the patients (Table 2).

As to possible clinical factors that affect patients’ BCAA adherence, we detected male sex, younger age, distribution of etiologies of liver cirrhosis, lower incidence of previous hepatic cancer, higher platelet count and lower serum ALT activities in the poor adherence group (Table 3). Among these factors, only male sex was also a possible unfavorable outcome marker (Table 2), but other factors were rather favorable or had no significance (e.g. cause of liver cirrhosis) for patients’ outcome (Table 2). Such observation suggests that particular caution should be paid for drug adherence in male cirrhotics.

The limitation of such studies on advanced liver cirrhosis is the possibility that earlier development of events shortly after the start of the study influenced treatment adherence. To address this concern, we additionally performed analysis after excluding the patients who developed any event within 6 months of the study, and the cumulative event-free survival rate was still significantly higher for the good adherence group than that for the poor adherence group (hazard ratio = 1.57, *P* = 0.0043), as was the case with the analysis on the whole analysis set.

In conclusion, higher treatment adherence for BCAA is considered to be associated with an improved serum albumin level, thereby leading to improved patient outcome. These results indicate the importance of patient instruction for the adequate use of BCAA granules.
